# Diagnostic biomarkers for active tuberculosis: progress and challenges

**DOI:** 10.15252/emmm.202114088

**Published:** 2022-10-31

**Authors:** Betânia M F Nogueira, Sonya Krishnan, Beatriz Barreto‐Duarte, Mariana Araújo‐Pereira, Artur T L Queiroz, Jerrold J Ellner, Padmini Salgame, Thomas J Scriba, Timothy R Sterling, Amita Gupta, Bruno B Andrade

**Affiliations:** ^1^ Programa de Pós‐graduação em Ciências da Saúde Universidade Federal da Bahia Salvador Brazil; ^2^ Instituto Couto Maia Salvador Brazil; ^3^ Multinational Organization Network Sponsoring Translational and Epidemiological Research (MONSTER) Initiative Salvador Brazil; ^4^ Division of Infectious Diseases, Department of Medicine Johns Hopkins University School of Medicine Baltimore MD USA; ^5^ Curso de Medicina Universidade Salvador (UNIFACS) Salvador Brazil; ^6^ Programa de Pós‐Graduação em Clínica Médica Universidade Federal do Rio de Janeiro Rio de Janeiro Brazil; ^7^ Laboratório de Inflamação e Biomarcadores, Instituto Gonçalo Moniz Fundação Oswaldo Cruz Salvador Brazil; ^8^ Faculdade de Medicina Universidade Federal da Bahia Salvador Brazil; ^9^ Center of Data and Knowledge Integration for Health (CIDACS), Instituto Gonçalo Moniz Fundação Oswaldo Cruz Salvador Brazil; ^10^ Department of Medicine, Centre for Emerging Pathogens Rutgers‐New Jersey Medical School Newark NJ USA; ^11^ South African Tuberculosis Vaccine Initiative and Institute of Infectious Disease and Molecular Medicine, Division of Immunology, Department of Pathology University of Cape Town Cape Town South Africa; ^12^ Division of Infectious Diseases, Department of Medicine Vanderbilt University Medical Center Nashville TN USA; ^13^ Curso de Medicina Faculdade de Tecnologia e Ciências (FTC) Salvador Brazil; ^14^ Curso de Medicina Escola Bahiana de Medicina e Saúde Pública (EBMSP) Salvador Brazil

**Keywords:** active TB, biomarkers, diagnostic biomarkers, tuberculosis, Biomarkers, Microbiology, Virology & Host Pathogen Interaction

## Abstract

Tuberculosis (TB) is a leading cause of morbidity and mortality from a single infectious agent, despite being preventable and curable. Early and accurate diagnosis of active TB is critical to both enhance patient care, improve patient outcomes, and break *Mycobacterium tuberculosis* (*Mtb*) transmission cycles. In 2020 an estimated 9.9 million people fell ill from *Mtb*, but only a little over half (5.8 million) received an active TB diagnosis and treatment. The World Health Organization has proposed target product profiles for biomarker‐ or biosignature‐based diagnostics using point‐of‐care tests from easily accessible specimens such as urine or blood. Here we review and summarize progress made in the development of pathogen‐ and host‐based biomarkers for active TB diagnosis. We describe several unique patient populations that have posed challenges to development of a universal diagnostic TB biomarker, such as people living with HIV, extrapulmonary TB, and children. We also review additional limitations to widespread validation and utilization of published biomarkers. We conclude with proposed solutions to enhance TB diagnostic biomarker validation and uptake.

## Graphical abstract

In this review, B. Andrade and colleagues discuss progress and challenges in the development of diagnostic biomarkers for active tuberculosis and propose potential solutions to improve tuberculosis biomarker validation and implementation.

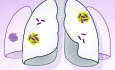

**Glossary**
Active tuberculosis: Disease occurred in individuals infected with *Mycobacterium tuberculosis*.
Antibody: Protein produced in response to antigens.
Antigen: Foreign substance which induces immune response (i.e., proteins from microbial surface).
Biomarker: A measurable marker that indicates a biological state or condition.
Biosignature: A group of biomarkers that indicate a specific biological state or condition.
Diagnostic biomarker: A marker that detects the presence of a disease or condition.
External validation: To be able to generalize the findings of a study to other contexts.
Extrapulmonary tuberculosis: Active tuberculosis that occurs in organs other that the lungs.
Internal validation: The extent to which a study results represent the truth in the population studied.
Cytokine: Molecules that play important roles in immune responses and cell–cell communication.
Host: The individual who becomes infected by an infectious agent.
*Mycobacterium tuberculosis*
: A species of mycobacteria responsible for causing tuberculosis.
Metabolomics: The study of metabolites and its behavior in different body processes.
Multiomics: Biologic analysis that includes different omics (i.e., proteomics, transcriptomics, etc.).
Omics: Study of biological molecules in different levels that describes the dynamics and function of an organism.
Pathogen: An agent that can cause disease.
Point‐of‐care: Testing that is performed near or at the site of a patient.
Proteomics: Study of proteins and how they relate with body processes.
Pulmonary tuberculosis: Tuberculosis disease that affects the lungs.
Target product profile: Desired characteristics of a target product.
Transcriptomics: The study of transcriptome and its behavior in different body processes.
Tuberculosis infection: Presence of *Mycobaterium tuberculosis* in the body that generates immune response, but not symptoms (active disease).

## Introduction

Tuberculosis (TB) is a communicable disease spread by inhalation of *Mycobacterium tuberculosis* (*Mtb*), primarily leading to infection of the lungs but also other sites. Approximately one‐quarter of the world's population is estimated to be infected with *Mtb* (Houben & Dodd, 2016), but in most individuals it exists in the form of tuberculosis infection (TBI), lying dormant through host immunologic control (Cohen *et al*, 2019; Shah & Dorman, 2021). In a subset of individuals, active disease develops when *Mtb* overcomes the host immune response, leading to increased bacterial replication and inflammation, which if left untreated can progress to critical illness and ultimately death. Until the advent of the COVID‐19 pandemic, active TB (ATB) was the leading infectious cause of mortality worldwide and it remains a major global public health concern.

While TB is both preventable and curable, the COVID‐19 pandemic has led to significant setbacks, with health service disruptions leading to an estimated increase in TB incidence by 5–15% over the next 5 years based on mathematical models (WHO, 2021c). Furthermore, repercussions of the pandemic are already evident, with deaths from TB increasing by 100,000 individuals from 2019 to 2020 to 1.3 million deaths estimated (Hogan *et al*, 2020; McQuaid *et al*, 2020; The Global Fund, 2021; WHO, 2021c; Pai *et al*, 2022). The COVID‐19 pandemic severely limited TB case detection and revealed the dire need for development of new TB diagnostic tools (WHO, 2021d). Point‐of‐care diagnostic tools developed during the COVID‐19 pandemic highlighted the shortcomings of ineffective testing which can lead to unacceptable delays in diagnoses and treatment with increased risk of transmission. As with COVID‐19, easy access to TB diagnostics remains challenging, particularly in highly endemic areas (WHO, 2021b). The use of real‐time surveillance data and large research networks that allow access to large and diverse datasets during the COVID‐19 pandemic may be applied to expedite TB diagnosis research.

Although identification and diagnosis of ATB in the infected individual is critical, challenges exist. For decades, smear microscopy to evaluate for acid fast bacilli and bacterial culture were the primary methods for diagnosis and confirmation of ATB. More recently, nucleic acid amplification of *Mtb* through tests such as GeneXpert MTB/RIF and GeneXpert MTB/RIF Ultra have also been added to the arsenal of diagnostics. Although culture and GeneXpert have high sensitivity and specificity in smear‐positive cases, they have diminished diagnostic accuracy in individuals with smear‐negative disease, people living with HIV (PLWH), children, and extrapulmonary TB (EPTB).^5^ Furthermore, culture is slow to yield results and GeneXpert MTB/RIF is costly and requires significant infrastructure to implement, limiting its widespread use. These methods have further limitations in monitoring response to treatment, which currently involves a minimum of 4–6 months of therapy. Given the underperformance and challenges of currently implemented TB diagnostics, new solutions to improve diagnosis and care are urgently required to meet 2030 World Health Organization (WHO) End TB targets of decreasing TB incidence by 80% and TB deaths by 90% compared to 2015 levels.^3^

**Figure 1:**
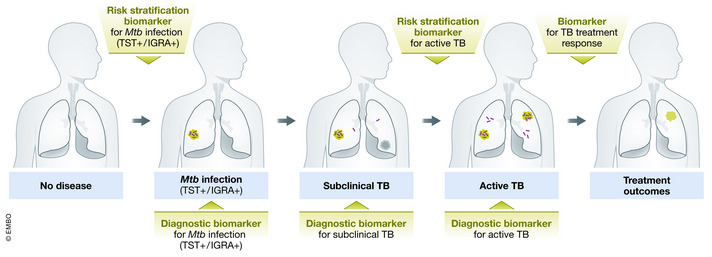
Wide range of available biomarkers within the spectrum of TB Abbreviations: *Mtb*, *Mycobacterium tuberculosis*; TB, tuberculosis; TST^+^, tuberculin skin test positive; IGRA^+^, interferon‐gamma release assay positive.

The use of biomarkers represents one such strategy. A biomarker is “a defined characteristic that is measured as an indicator of normal biological processes, pathogenic processes or responses to an exposure or intervention” (FDA‐NIH Biomarker Working Group, 2016). Within the field of TB, both pathogen and host biomarkers have been extensively explored. Pathogen‐based biomarkers include both DNA and antigen detection. A wide variety of host‐based biomarkers exist, including hematologic markers, antibody response to antigen, cytokines and chemokines, RNA, other proteins, metabolites, and signatures that combine multiple markers, some of which have been identified by unbiased “omics” discovery approaches. The application of these biomarkers can be broken down by their utility as diagnostic tests, including for point‐of‐care testing, prognostic tests for risk of progression to incident ATB, and treatment response markers that may predict TB treatment outcomes (Fig 1). The WHO has included the development of a diagnostic biomarker or triage biomarker among the high‐priority target product profiles (TPPs; WHO, 2014). Ideally a biomarker would be low cost, obtained from a readily accessible sample, such as blood or urine, and the equipment used to measure the biomarker would be easy to use and point‐of‐care. While there has been success in clinical use of pathogen‐based biomarkers in the form of Cepheid GeneXpert and Urine Lipoarabinomannan (LAM), host‐based biomarkers are in less advanced stages of development.

Numerous reviews have recently been published, summarizing available ATB biomarkers in the literature (MacLean *et al*, 2019; Yong *et al*, 2019; Wykowski *et al*, 2021). The goal of this review is to explore currently available diagnostic TB biomarkers, to lend our perspective on challenges to widespread implementation of these biomarkers, and to offer possible solutions to move forward with their usage in the future.

## Currently available ATB diagnostic biomarkers

**Figure 2:**
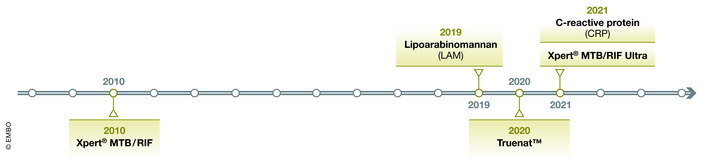
Timeline of the TB diagnostic biomarkers endorsed by the World Health Organization

**Figure 3:**
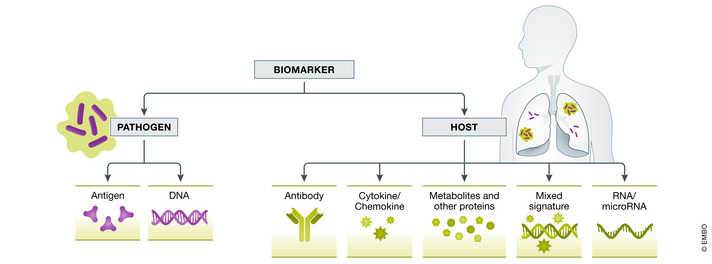
Spectrum of host and pathogen biomarkers that have been widely studied as TB diagnostics

There are a wide range of discovered diagnostic TB biomarkers specific either to the host or the pathogen. These biomarkers have had varying degrees of success in meeting WHO TPP criteria. These criteria include a goal optimal sensitivity of ≥ 98% in smear‐positive, culture‐positive pulmonary TB (PTB), ≥ 68% in smear‐negative, culture‐positive adults, and an overall pooled sensitivity of ≥ 80% in adults with HIV. An optimal TPP diagnostic sensitivity of ≥ 85% in lymph node‐based extrapulmonary TB (EPTB) versus ≥ 80% in cerebral spinal fluid is recommended. Finally, for microbiologically confirmed TB, the goal TTP sensitivity is ≥ 66% in childhood intrathoracic TB. Despite the varied sensitivity for these TB disease states, an all‐encompassing goal TTP specificity of ≥ 98% is recommended (WHO, 2014). A timeline of the TB diagnostic biomarkers endorsed by the WHO can be found in Fig 2. Using the optimal TTP diagnostic criteria outlined by the WHO as a benchmark, in this section, we will review some of the most promising diagnostic biomarkers (Fig 3).

### Pathogen biomarkers

GeneXpert MTB/RIF and LAM are the most studied pathogen‐specific biomarkers and are currently commercially available. GeneXpert MTB/RIF is an automated polymerase chain reaction (PCR) test used to rapidly diagnose ATB and detect rifampicin resistance. Unlike conventional nucleic acid amplification tests, it is based on a single cartridge system that can both amplify and detect PCR in about 2 h using automated assays, thus requiring minimal training and biosafety measures (WHO, 2013). The limit of *Mtb* detection of GeneXpert was subsequently improved with the development of GeneXpert MTB/RIF Ultra test which is rapidly becoming the diagnostic gold standard (Dorman *et al*, 2018). Both GeneXpert MTB/RIF and GeneXpert MTB/RIF Ultra are recommended by the WHO for ATB diagnosis. Despite their excellent pooled sensitivity in smear‐positive samples (98%), TB detection is low in paucibacillary specimens (68%), particularly in PLWH (80%), children (66%) and in extrapulmonary samples (79%; WHO, 2013; Kohli *et al*, 2018; Osei Sekyere *et al*, 2019; Zar *et al*, 2019). Furthermore, GeneXpert MTB/RIF may remain positive for a prolonged period after development of ATB, thus the reliability of GeneXpert MTB/RIF‐positive results in patients with a history of TB and concern for relapsed disease remains unknown (Theron *et al*, 2016; Costantini *et al*, 2020). Finally, the technology remains expensive and requires a constant source of electricity, limiting its use in highly endemic resource‐limited settings (Nalugwa *et al*, 2020). GeneXpert MTB/RIF has represented an important advance in TB diagnosis facilitating earlier diagnosis and treatment where it is available (Ershova *et al*, 2020; Tamirat *et al*, 2022). It has, however, not been able to increase TB detection worldwide as initially expected, in part due to continued treatment of suspected TB based on clinical criteria rather than a diagnostic test (Walzl *et al*, 2018). Recently, the WHO has endorsed Truenat as another molecular test to be used in ATB and rifampicin resistance diagnosis. Different studies have found Truenat to have a similar accuracy compared to GeneXpert MTB/RIF (Nikam *et al*, 2014; Penn‐Nicholson *et al*, 2021; Ngangue *et al*, 2022), though Truenat has the advantage of being supplied by a battery‐powered system, thus requiring less infrastructure (Lee *et al*, 2019).

Lipoarabinomannan is a component of the mycobacterial cell wall that is released from metabolically active bacterial cells and excreted in host urine. While it offers a low cost, highly specific antigen that can be detected quickly, its low sensitivity in individuals other than severely immunocompromised PLWH limits its use (Lawn *et al*, 2012). The WHO recommends the use of AlereLAM only in PLWH who are severely ill or with a CD4 count lower than 100 cells/mm^3^ (Engel & Mwaura, 2019). The sensitivity of the test has been further improved with the development of FujiLAM which detects urine LAM concentrations 30 times lower than AlereLAM through the use of innovative assays and reagents. This allows for improved sensitivity for TB detection in HIV‐negative individuals and PLWH with higher CD4 counts (Sigal *et al*, 2018). A recent meta‐analysis found that FujiLAM had good accuracy in diagnosing ATB in adults, with a sensitivity and specificity of 0.70 and 0.93, respectively (Li *et al*, 2021).

### Host biomarkers

#### Antibody response

Antibodies are frequently used as biomarkers in infectious diseases because they are generally inexpensive and simple to test. Antigen‐specific antibodies against *Mtb* as tests for pulmonary or extrapulmonary TB are the most studied biomarker category. However, they have not proven useful in differentiating between asymptomatic *Mtb* infection and ATB, and have poor diagnostic accuracy due to their low avidity to the surface antigen (Perley *et al*, 2014; Correia‐Neves *et al*, 2019), which results in the highly variable sensitivity and specificity found in numerous studies (MacLean *et al*, 2019; Yong *et al*, 2019). In fact, the WHO has advised against the use of the currently commercially available antibody tests for TB (WHO, 2011). Given the poor accuracy of antibody assays, different strategies to increase TB detection have been proposed such as the simultaneous assessment of multiple antigen‐specific antibodies (Rekha *et al*, 2011) or the search for more specific antigens or epitopes (Sigal *et al*, 2018). Despite these alternative strategies, it does not currently appear that antibodies will have an important role in TB diagnosis in the near future.

#### Cytokine response

A wide range of cytokines have been studied as biomarkers for TB detection with more promising results. For instance, measurement of Interferon‐Gamma (IFN‐γ) release following blood stimulation with Mtb‐specific antigens through the Interferon Gamma Release Assay (IGRA) represents an important step in the diagnosis of current or prior Mtb infection, although it is not accurate for ATB diagnosis. IGRA demonstrates improved sensitivity and specificity in those with Bacille Calmette‐Guerin (BCG) vaccination and PLWH when compared to the Tuberculin Skin Test (TST) that involves injection of tuberculin antigen in the forearm, followed by assessment for any local reaction (lump). Despite the accuracy for diagnosis of TB infection, IGRA use is still limited by cost and challenging logistics needed for blood collection, transportation, and sample processing (Yong *et al*, 2019).

For the diagnosis of ATB, a combination of select cytokines to form specific biosignatures has shown sufficient accuracy (Chegou *et al*, 2016, 2018; Jacobs *et al*, 2016; MacLean *et al*, 2019) and currently represents a leading strategy, although in most cases the studies still lack validation in areas with differing TB prevalence and from geographically distinct populations (MacLean *et al*, 2019). African studies using cytokine biosignatures met the criteria set in the WHO TPPs for the diagnosis of pulmonary TB, but had significant variation in accuracy between smear‐negative and ‐positive cases (Chegou *et al*, 2016, 2018). In many cases, cytokines are combined with other proteins to form more accurate biosignatures. Chegou *et al* identified a seven‐marker protein signature that was accurate regardless of HIV status or ethnicity in Africa. The findings are yet to be validated in other ethnicities outside of Africa (Chegou *et al*, 2016).

#### Biosignatures discovered through omics

With the development of high‐throughput methodologies, increasingly an “omics” approach, with complete identification and characterization of a class of biological molecules, has been evaluated to identify a biosignature for TB disease. The term “omics” includes genomics, transcriptomics, proteomics, and metabolomics, providing a comprehensive study of biomarkers in a single step. Genomics provides genetic information, while transcriptomics identifies gene expression patterns, proteomics reveals protein products during disease, and metabolomics characterizes the metabolism of an individual during TB disease (Yong *et al*, 2019). While the “omics” approach is promising, it requires costly technological infrastructure and analytic pipeline infrastructure technology which may be unavailable in most settings. It has been useful to improve understanding of the host response to TB, ultimately leading to the identification of relevant markers, but the exact use and value of omics‐discovered biomarkers in TB diagnosis is still to be determined (Yong *et al*, 2019).

##### Transcriptomics

In TB research, transcriptomics studies that have defined blood gene expression signatures with diagnostic utility are the most advanced. A recent systematic review and meta‐analysis found that 17 transcriptomic signatures met at least one TPP minimum performance criterion for TB diagnosis and three were validated in clinically relevant cohorts (Mulenga *et al*, 2020). RISK11, an 11‐gene transcriptomic signature, performed well as a screening test for symptomatic ATB (Zak *et al*, 2016; Darboe *et al*, 2019; Mendelsohn *et al*, 2021), but performance for asymptomatic (sub‐clinical) TB was significantly lower (Scriba *et al*, 2021). A follow‐up study that compared eight parsimonious signatures showed a similar picture, where diagnostic performance for symptomatic TB disease was mostly excellent, but performance for sub‐clinical disease was significantly lower (Mendelsohn *et al*, 2022). A large study using publicly available datasets used different methods to identify expressed genes to distinguish ATB from controls and found a 380‐gene meta‐signature that can be further explored (Blankley *et al*, 2016a). In light of the high prevalence of subclinical disease (Frascella *et al*, 2021) excellent diagnostic performance in subclinical TB is critical to improve case‐finding strategies.

RISK11 has also been evaluated prospectively in a clinical trial to identify those at risk for incident TB. Prognostic performance was excellent for incident TB occurring 6–9 months after biomarker testing, but waned significantly thereafter (Scriba *et al*, 2021). Similar prognostic performance, with limited duration beyond 9 months was also reported for eight parsimonious signatures (Mendelsohn *et al*, 2022). The CORTIS study also assessed efficacy of RISK11‐guided TB preventive therapy in HIV‐negative adults in a high TB prevalence area, but found no effect on TB risk over the 15‐month follow‐up period (Scriba *et al*, 2021). The authors argue that this lack of efficacy for incident TB may have been influenced by the therapeutic regimen used or reinfection cases. Interestingly, transient efficacy of tuberculosis preventive treatment (TPT) through 9 months was noted among fully adherent participants. In 2016, Sweeney *et al* developed a three‐gene signature for diagnosis of pulmonary TB (PTB), which has now been developed into a prototype MTB‐Host Response cartridge (Cepheid) that measures the signature on capillary blood collected from a fingerstick (Sweeney *et al*, 2016). This prototype MTB‐Host Response test has been evaluated as a triage test and met the minimal TPP criteria using Xpert Ultra as the reference standard in a prospective cohort (Sutherland *et al*, 2021).

##### Metabolomics

Another developing area of study is the use of metabolomics in the identification of biomarkers related to TB. Although in early stages of development, metabolomic biomarkers may offer an unique application to routine clinical samples, as measurements may be performed on non‐invasive samples, like urine, with minimal sample preparation and offer the potential for development of cheaper equipment (Luies *et al*, 2017). A metabolomic profile was evaluated in a prospective multisite study across four African countries, in serum and plasma from HIV‐negative, TB‐exposed individuals to evaluate risk of TB disease. The authors identified a metabolic biosignature with a performance of 69% sensitivity and 75% specificity within 5 months of diagnosis, thus identifying subclinical stages of TB disease. As with most biomarker studies, these findings remain to be validated outside of Africa, but may offer promising tools for TB control through early treatment in a pre‐symptomatic phase (Weiner *et al*, 2018). Other studies have focused on the discovery of new metabolites to diagnose TB. Cho *et al* (2020) used a targeted approach emphasizing non‐lipid metabolites that identified ratios between amino‐acids that were able to differentiate between active TB, TB infection, and healthy controls. Conde *et al* (2021) evaluated amino‐acids signatures and detected a signature that met the WHO TPP recommendation for a triage test to rule‐out active TB. Concentrations of biomarkers related to iron pathways have been tested in a large cohort and were included in a prediction model with sufficient accuracy to diagnose ATB and correlated with the degree of lung damage and bacillary load (Dai *et al*, 2019).

##### Proteomics

Proteomics is also a growing area of research in the TB field. The development of improved methods in quantitative proteomics (Mass Spectrometry‐based techniques) has increased the relevance of proteins as a system‐level approach (Banaei‐Esfahani *et al*, 2017) that complements both genomics and traditional biochemical techniques. So far, this method has aided in better understanding the complex interaction between *Mtb* and the host, but signatures that meet TPPs and are useful as diagnostic tests are yet to be found. A recent study has shed light on unique processes in lipid transport, iron assimilation, acute‐phase response, and inflammation in individuals with ATB, compared to their contacts with TBI (Mateos *et al*, 2020). Many discovery studies have had promising candidates with variable accuracy (Liu *et al*, 2013; Banaei‐Esfahani *et al*, 2017; Mateos *et al*, 2020). A six marker signature including SYWC, kallistatin, complement C9, gelsolin, testican‐2, and aldolase C was found to have 90% sensitivity and 80% specificity in ATB diagnosis, thus meeting the screening TPP but not the diagnostic TPP (De Groote *et al*, 2017). Some proteomic profiles were even able to predict progression to active TB prior to the diagnosis. Garay‐Baquero *et al* (2020) have identified a proteomic TB risk signature that was able to predict progression to incident TB within a year of diagnosis, although it did not meet the WHO TPP. In PLWH, panels of 5 to 12 proteins were able to predict ATB up to 2 years before diagnosis (Singer *et al*, 2022).

##### MicroRNAomics

MicroRNAs (miRNAs) are small, noncoding RNA that were first thought to have no biological role. More recently, research has shown that miRNAs are involved in cellular processes and thus have altered expression during various diseases, such as TB (Ruiz‐Tagle *et al*, 2020). Different miRNA expression profiles have been found according to *Mtb* strain, drug resistance, response to treatment, or stages of TB disease, turning them into potential biomarkers (Ren *et al*, 2015; Zheng *et al*, 2015; Wang *et al*, 2018). Nevertheless, to date, the studies lack validation and their findings cannot be compared due to relevant methodological differences in processes such as RNA extraction or data analysis (Sabir *et al*, 2018; Ruiz‐Tagle *et al*, 2020).

##### Multi‐omics

The multi‐omics approach includes simultaneously evaluating different types of biomarkers, offering a comprehensive view of genotype–phenotype relationships in TB pathogenesis, and possibly generating accurate biomarkers. Besides being comprehensive, it is an unbiased method (Ahamad *et al*, 2022). A recent study used three different modalities and integrated omics analysis comparing PLWH with and without incident TB. The authors found that 11 miRNAs and three serum cytokines (tumor necrosis factor, interferon‐γ‐inducible protein‐10/CXCL10, and macrophage‐derived chemokine/CCL22) were differentially expressed in incident TB cases. A decision‐tree algorithm using multi‐omics data found that gamma‐glutamylthreonine and hsa‐miR‐215‐5p had the ability to accurately discriminate incident TB cases from controls with a high degree of accuracy (area under the curve [AUC] of 0.965; Krishnan *et al*, 2021). There are added complexities to measuring combinatorial biomarkers based on different types of molecules, making this approach more challenging and expensive.

## Challenges with development of biomarkers in special populations

Heterogenous populations, even among those with ATB, pose a barrier to further development and widespread implementation of published biomarkers. Subgroups within TB that span the spectrum of infection and disease create unique biosignatures or cause biomarkers to have varying sensitivities or specificities that are often below WHO targets.

### Extrapulmonary TB

Extrapulmonary TB represents nearly 20% of all TB cases in developing countries (WHO, 2021c), although this manifestation of TB is likely under‐recognized. The diagnosis of EPTB is often challenging as it requires invasive procedures to obtain tissue samples and the sensitivity of Gene Xpert MTB/RIF is lower than in sputum, though it varies across extrapulmonary specimen sites (Denkinger *et al*, 2014; Kohli *et al*, 2018). Most TB biomarker studies primarily include individuals with PTB, therefore limited information exists on the performance of biomarkers in persons with EPTB. Studies to date have had mixed findings, small sample sizes, and lack of validation in other populations (Fortún *et al*, 2014). Most evaluate pleural TB only, excluding relevant and hard‐to‐diagnose forms of EPTB, such as TB meningitis. A study that evaluated levels of interferon‐gamma, chemokine ligand 9, mannose‐binding lectin (MBL), tumor marker Ca‐125, and adenosine deaminase in both PTB and EPTB found that only MBL displayed different levels among PTB and EPTB cases (Fortún *et al*, 2014). Kathamuthu *et al* (2020) found that some matrix metalloproteinases (MMPs) and tissue inhibitors of metalloproteinase (TIMPs) were able to differentiate PTB and EPTB from infected and uninfected controls. Similarly, Chakrabarty *et al* (2019) identified a panel of host and *Mtb* miRNAs differentially expressed in serum from pulmonary TB and EPTB cases. A study done in Brazil found that cell activation markers (CD38, HLADR, and Ki67) in Mtb‐specific CD4^+^ T cells distinguished EPTB from TBI and PTB, regardless of HIV infection status (Silveira‐Mattos *et al*, 2020).

One of the major challenges in finding accurate biomarkers to diagnose EPTB is the heterogeneity of the later. For instance, one study identified a 380 meta‐signature that could differentiate ATB from healthy controls, though the main genes of the signature had lower sensitivity for EPTB compared to PTB and the magnitude of the transcriptional response varied according to the disease site and presence of symptoms (Blankley *et al*, 2016b).

### Pregnant women

Few diagnostic TB biomarkers studies have been completed in pregnant women, a population that has both increased risk of developing TB and heightened risk of morbidity to both the mother and fetus if not diagnosed promptly (Mathad & Gupta, 2012; Jonsson *et al*, 2020; Miele *et al*, 2020). Pregnancy induces altered immune function with accompanied immune suppression which appears to impact the clinical presentation of active TB and the sensitivity of diagnostics (Mor & Cardenas, 2010; Getahun *et al*, 2012). Studies have demonstrated that interferon gamma release assay positivity is impacted for pregnant women with TB infection (Mathad *et al*, 2014; LaCourse *et al*, 2017; Bhosale *et al*, 2021; Weinberg *et al*, 2021) but little is known regarding the performance of diagnostic biomarkers of active TB in pregnancy and future studies are needed.

### Children

Pediatric TB is difficult to diagnose as children frequently have paucibacillary disease. Pediatric TB can progress rapidly and often leads to severe disease if left untreated. Clinical and radiological findings are often nonspecific, and sputum samples are hard to obtain. Once a sample has been obtained, microbiological confirmation is made in only up to 40% of cases, since TB in children is usually paucibacillary (Gjøen *et al*, 2017; Nicol & Zar, 2020). TB biomarker studies in children share similar limitations: lack of validation in different populations and typically small sample sizes with lack of culture‐confirmed cases. Given the challenge to identify a large cohort of children with microbiologically confirmed TB, often the true estimate of test accuracy is difficult to determine. Despite these difficulties, it is critical to include children in diagnostic studies with biomarkers, as children with TB have been shown to have different immune response profiles, compared to adults (Tornheim *et al*, 2020).

Studies have identified promising biomarkers that require further exploration. A T‐cell activation marker assay has shown 83% sensitivity and 97% specificity in a small prospective proof‐of‐concept study that evaluated 113 children, of whom 18 had culture‐confirmed TB (Portevin *et al*, 2014). A cross‐sectional study conducted in India comparing blood samples of 47 children with TB to healthy controls and symptomatic non‐TB cases identified a 7‐ and a 10‐transcript blood signature with AUC of 0.94 (95%CI, 0.88–1.00) to identify ATB (Gjøen *et al*, 2017). Recently, researchers in India evaluated an integrated blood‐based metabolomic and transcriptomic signature in children with confirmed TB compared to uninfected household contact children, and identified N‐acetylneuraminate, quinolinate, and pyridoxate as candidate biomarkers for ATB identification (Dutta *et al*, 2020). Also in India, a biosignature consisting of C‐reactive protein, MMP‐7, and lipopolysaccharide‐binding protein (LBP) was able to accurately distinguish between pediatric PTB, EPTB, and healthy controls (Albuquerque *et al*, 2019). A larger multi‐center African study identified a 51‐transcript RNA signature from children with culture confirmed or clinically suspected TB. The sensitivity and specificity were 83 and 84% for microbiologically proven TB, although they varied significantly among highly probable, probable, or possible cases of TB (Anderson *et al*, 2014).

### Individuals with low bacillary burden

Smear‐negative TB represents a significant portion of all TB cases. A cross‐sectional study in New York of 796 HIV‐negative patients with pulmonary TB, found that 15% of cases were sputum smear and culture negative. These patients had a significantly lower proportion of symptoms and cavitation on imaging compared with patients with culture‐positive disease (Nguyen *et al*, 2019). The proportion of smear‐negative patients may be even higher in PLWH, reaching up to 65% (Campos *et al*, 2016). There are few studies comparing biomarkers between smear‐positive or smear‐negative TB, though many studies that analyzed biomarkers in PTB found similar biomarkers in both smear‐positive and negative. However, in general, these studies identified lower diagnostic accuracy for biomarkers in smear‐negative pulmonary TB, compared to smear‐positive PTB (Chegou *et al*, 2016, 2018).

An Africa‐wide study analyzed a seven‐biomarker protein signature in both smear‐positive and negative cases that included CRP, transthyretin, interferon‐γ (IFN‐γ), complement factor H, apolipoprotein‐A1, inducible protein 10 and serum amyloid A, for the diagnosis of PTB. This signature classified 74% of patients who were smear‐negative but culture‐positive, and 67% of patients who were both smear and culture‐negative, significantly less than when compared to the accuracy for recognition of smear‐positive (88%) and culture‐positive cases (91%) in the same study (Chegou *et al*, 2018). Also in Africa, Chegou *et al* (2016) found that the most accurate biosignature for the diagnosis of smear‐positive TB was IFN‐γ, transforming growth factor‐α (TGF‐α), interleukin‐1α (IL‐1α), MMP‐2, epidermal growth factor (EGF), and antigen‐specific levels of vascular endothelial growth factor (VEGF) and TGF‐α, with 75.7% sensitivity and 80% specificity, whereas the most accurate biosignature for the diagnosis of smear‐negative TB was IFN‐γ, IFN‐α, sCD40L, IL‐1α, MMP‐2, MMP‐9, and IFN‐α2 with 60% sensitivity and 70.8% specificity. Liu *et al* (2013) found that a serum proteomic profile could differentiate sputum smear‐negative and smear‐positive TB patients from controls with reasonable accuracy (81.59%), sensitivity (78.57%), and specificity (84.62%).

While the performance of biomarkers in smear‐negative TB is limited, their accuracy as diagnostic tools may improve when combined with clinical features to generate a composite scoring system. Field studies to evaluate the clinical characteristics of smear‐negative TB with biomarkers may be limited in the absence of a gold standard diagnostic.

### TB‐HIV co‐infection

The high frequency of smear‐negative pulmonary TB and EPTB, combined with atypical clinical presentations hinders accurate diagnosis of TB in PLWH (Getahun *et al*, 2007; Sama *et al*, 2016; Teixeira *et al*, 2018). Co‐infection with TB‐HIV is critically important to diagnose and treat as it accounts for approximately 8% of ATB cases worldwide, with a disproportionately high mortality (WHO, 2021c). Nevertheless, most TB biomarker discovery studies have focused on HIV‐uninfected populations or only include a small sample of PLWH, with low power to determine differences in biomarkers in this group. Accuracy may vary depending on the extent of immunodeficiency related to HIV infection, therefore studies in this population will require a large and diverse population of PLWH included.

The most recent version of the WHO guidelines on TB (WHO, 2021a) has included C‐reactive protein (CRP) as a screening marker in ambulatory PLWH. The recommendation was based on the findings of a meta‐analysis that high CRP had a similar sensitivity and higher specificity than the WHO‐recommended four‐symptom screen alone (Dhana *et al*, 2022). CRP has the advantage of being a low‐cost point‐of care test that can be done on capillary blood. Nevertheless, it does not meet TPP for specificity, particularly in inpatients or PLWH who are on antiretrovirals. Other circulating infectious agents such as COVID‐19 that raise CRP may further reduce the specificity of CRP.

Achkar *et al* (2015) identified several host serum proteins that differed among HIV‐positive and ‐negative patients but showed good accuracy in both groups to differentiate ATB from individuals with other respiratory diseases or asymptomatic individuals with *Mtb* infection. A recent study in South Africa identified urine immunological biomarkers that were able to distinguish PTB from other respiratory diseases in PLWH with adequate accuracy, although it varied according to CD4 levels (Eribo *et al*, 2020).

Many transcriptomic signatures for TB diagnosis lack evaluation in PLWH. For example, a prospective, multicenter cohort study aimed to prospectively validate six transcriptomic signatures developed within the cohort, and an additional three that were previously published. The signatures had insufficient accuracy for diagnosis of TB and were limited by a low prevalence of HIV (only 7%); in addition, the study was conducted in an area with low TB burden (Hoang *et al*, 2021). On the other hand, a case–control study that took place in South Africa and Malawi found blood transcriptional signatures that had similar accuracy in both HIV‐negative and ‐positive cohorts, though slightly lower in PLWH (Kaforou *et al*, 2013).

A recent observational study evaluated the diagnostic and prognostic performance of transcriptomic signature (RISK11) to identify ATB and risk of progression to incident TB within 15 months in ambulatory PLWH. RISK11's performance approached, but did not meet, WHO TPP benchmarks for screening and diagnostic tests for TB, although the small number of participants with TB limited the precision of these estimates. The effect of antiretroviral therapy, isoniazid preventive therapy, CD4 cell count, and HIV plasma viral load, on this signature could not be evaluated due to the small number of TB cases (Mendelsohn *et al*, 2021). Eight parsimonious TB transcriptomic signatures were validated in HIV‐uninfected and HIV‐infected adults in South Africa. No signature met the WHO TPP benchmarks for subclinical TB, though most met the TPP for symptomatic disease in the HIV‐uninfected population. Importantly, the signatures were found to be upregulated by viral infections and detectable viral load in PLWH, even in those without TB, suggesting one reason for reduced specificity of blood transcriptomic signatures in community studies (Mendelsohn *et al*, 2022).

Serological biomarkers were evaluated in serum samples (*n* = 404) from repositories managed by the Foundation for Innovative New Diagnostics (FIND) TB to investigate the influence of HIV co‐infection and host factors on serological biomarkers, using anti‐A60 IgG and IgA and CRP as markers of TB. The study found a high degree of variation and complexity in the relationships between biomarker distributions and host factors with lower sensitivity and specificity in HIV subpopulations, implying limited application of host factor screening in TB serodiagnostic algorithms in PLWH (Mohd Hanafiah *et al*, 2019).

## Challenges to validation and utilization of available biomarkers

**Figure 4:**
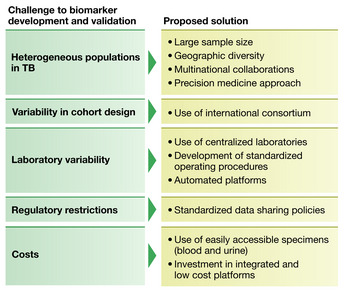
Challenges to biomarker development and proposed solutions

Following biomarker discovery and analytical validation, where the performance characteristics of biomarkers are established, biomarkers must be both internally and externally validated and their clinical utility must be established to reach widespread use (Ou *et al*, 2021). Internal validation occurs within the dataset where the biomarker was developed and can be assessed through statistical methodology, such as testing the sensitivity and specificity of a biomarker in a training and validation set or through bootstrapping (FDA‐NIH Biomarker Working Group, 2016; Ou *et al*, 2021). External validation evaluates the biomarker's performance in an independent cohort, ideally from diverse epidemiological and geographic backgrounds (Ilyin *et al*, 2004; FDA‐NIH Biomarker Working Group, 2016). Clinical utility, a form of external validation, is often demonstrated through prospective clinical studies or trials. Significant roadblocks at the steps of internal validation, external validation, and evaluation of clinical utility have hindered the development of a widely used TB biomarker. Furthermore, fitted models often show the highest diagnostic performance in the discovery dataset, but have diminished sensitivity and/or specificity in the external validation dataset. We will discuss the major limitations to these validation processes within TB (Fig 4).

### Heterogeneous populations

As described in the previous section, a large and diverse group of patients need to be well‐represented in studies. Furthermore, biomarker levels may vary across distinct ethnic populations and by TB burden, therefore studies need to include ethnically diverse populations and epidemiologically different scenarios (low, medium, and high TB burden areas). This highlights the need for significantly large sample sizes, and it can be particularly challenging to include some populations such as pregnant women, children or PLWH with varying degrees of immunosuppression.

### Variability in study design

While TB cohorts with biobanked specimens exist to allow for external validation, significant differences in the study design can create bias and limit validation. For example, a biomarker may be developed in an observational cohort of ATB where the controls are household contacts. In these controls, there would be an expected high prevalence of TST‐positive or IGRA‐positive individuals. Changes in performance characteristics can be encountered when the assay is in turn externally validated using a cohort that uses individuals who are TST or IGRA negative as controls. Alternatively, a study may use individuals with an alternative pathological process (e.g., other respiratory diseases) as the controls. While not surprising that a biomarker would have different performance characteristics in this wide variety of available controls, there is ultimately great variability in the definition of the control group across cohort studies in the literature. Furthermore, some observational cohorts available for external validation lack a control group. Additionally, cohorts are often designed to assess different primary outcomes and obtain different predictor variables or use alternate definitions for these outcomes and variables. Small cohort sample sizes with few outcomes can lead to underpowered studies, which can hinder the process of biomarker discovery as well as internal and external validation. Finally, true active TB case prevalence is better reflected in prospective cohort studies, whereas case–control studies often select for increased prevalence, introducing potential bias.

### Laboratory variability

Laboratory variability and potential measurement errors contribute significantly to further validation of promising published TB biomarkers (White, 2011). In general, TB biomarkers are often discovered on assays performed on a single platform, with a single institution performing the assay. When the time comes to externally validate, even if samples are shipped to the institution where the biomarker was initially discovered, significant variability in sample processing and storage in the new cohort can lead to measurement error and can affect biomarker results. Investigators may seek to evaluate the biomarker at an alternative institution or country, but even small differences in assay protocol, variability in assay measurement and analysis can also affect results. For example, a multi‐plex cytokine assay from the same company can have different thresholds of normal if measured on a platform from two different companies. Additionally, even if the same brand of assay and machine are used, laboratory technician performed calibration and validation can lead to disparate results.

### Regulatory restrictions

External validation of promising biomarkers across heterogenous and diverse cohorts is critical. Frequently, multinational collaborative projects face challenges in data sharing because each country has unique privacy laws, regulatory restrictions, and policies that govern export of human tissues and specimens. This can lead to limitations and delays in both data sharing and importation or exportation of specimens (Vlahou *et al*, 2021).

### Cost

The burden of TB is largely concentrated in low‐ and middle‐income countries (LMICs; WHO, 2021a). Even among WHO endorsed diagnostics, uptake of technology such as GeneXpert has been limited, in part due to burdensome cost (Nalugwa *et al*, 2020; Gotham *et al*, 2021). This slow uptake has occurred despite concessional pricing to reduce the burden of implementation. Similarly, for other TB diagnostic biomarkers, the assays, equipment, and technology used to discover biomarkers are costly, although high‐throughput platforms have led to some diminished cost over time (Lightbody *et al*, 2019). There is further cost for sample processing and storage of specimens. Thus, although promising diagnostic biomarkers may be developed, further investigation and implementation of these platforms may come at too high of a cost to LMICs. As a result, it is important that promising novel biomarkers can be developed into near‐point‐of‐care tests that can be manufactured and distributed at low cost, as outlined in the WHO TPP documents.

## Overcoming challenges and conclusions: our suggested path forward

Despite the abundance of published studies on TB biomarkers, there is still much knowledge to be gained, especially in TB infection and disease states where microbiological tests are less effective. In the field of TB diagnostics, this includes populations such as smear negative TB, EPTB, pediatrics, pregnant women, and PLWH. Furthermore, biomarkers may yet shed light on identification of those at risk of progression from asymptomatic *Mtb* infection to active TB and in monitoring treatment response during TB therapy. Moving forward, to minimize the challenges discussed above, we suggest development of biomarkers within international networks or consortia such as the Regional Prospective Observational Research for Tuberculosis (RePORT) International consortium with sites in Brazil, China, India, Indonesia, the Philippines, and South Africa (Hamilton *et al*, 2015; Geadas *et al*, 2017; van der Heijden *et al*, 2018). This allows for use of standardized cohorts, sample collection and storage procedures, as well as harmonized laboratory protocols. Additionally, investigation of biomarkers within international cohorts, includes the benefit of enabling analyses across epidemiologic and geographic heterogeneity in larger sample sizes and thus increased number of outcomes, which can lead to a better understanding of performance across infection and disease states. The larger number of outcomes allows for increased statistical power and better adjustment of potential confounders. Furthermore, use of a centralized laboratory or at a minimum standardized operating procedure for biomarker discovery and validation is critical to decreasing interassay and inter‐laboratory variability. While it may seem that this approach could stifle innovation, within standardized cohorts and consortiums (such as RePORT), site investigators do have the ability to design and propose sub‐studies through concept sheets, allowing for either site‐specific exploration of novel ideas or a request to test a novel biomarker on biobanked specimens that were collected at multiple sites and countries, but stored in similar conditions, thus decreasing site‐specific variability. Furthermore, the established structure of a consortium allows for expansion and inclusion of more difficult to recruit populations, such as pregnant women or TB meningitis, where it would otherwise potentially be difficult to enroll sufficient numbers at a single site. While not without limitations, the consortium structure does serve as a foundation whereby all investigators have the opportunity to expand the platform and equitably access stored specimen.

Given the biohazards and infrastructure required for sputum collection and microbiological confirmation of disease, an ideal TB diagnostic biomarker would be derived from an easily accessed sample (such as urine or fingerstick blood) and run on a low‐cost, point‐of‐care platform to allow widespread global uptake. As we move forward, we may ultimately find that a one diagnostic‐fits‐all approach may be difficult to achieve given the complexity and heterogeneity of TB. The application of a precision medicine approach to the field of TB, such as the tailored use of a specific biomarker in unique at‐risk subgroups, may ultimately allow for the highest diagnostic accuracy.

## Author contributions

**Betânia M F Nogueira:** Conceptualization; writing – original draft; writing – review and editing. **Sonya Krishnan:** Conceptualization; writing – original draft; writing – review and editing. **Beatriz Barreto‐Duarte:** Visualization; writing – original draft; writing – review and editing. **Mariana Araújo‐Pereira:** Visualization; writing – original draft; writing – review and editing. **Artur T L Queiroz:** Writing – review and editing. **Jerrold J Ellner:** Writing – review and editing. **Padmini Salgame:** Writing – review and editing. **Thomas J Scriba:** Writing – review and editing. **Timothy R Sterling:** Writing – review and editing. **Amita Gupta:** Conceptualization; writing – original draft; writing – review and editing. **Bruno B Andrade:** Conceptualization; supervision; writing – original draft; writing – review and editing.

## Disclosure and competing interests statement

The authors declare that they have no conflict of interest.

## For more information

- https://www.who.int/publications/i/item/WHO‐HTM‐TB‐2014.18
- https://www.who.int/publications/i/item/9789240029415

**Pending issues**

- Development and validation of a diagnostic biomarker that diagnoses TB disease in both pulmonary and extrapulmonary tuberculosis and in varying stages of immunosuppression in both adults and children.
- Development of a low‐cost, user friendly, point‐of‐care tuberculosis diagnostic test that uses easily to obtain specimen and is accurate across the spectrum of active TB disease.
- Improved sensitivity and specificity of TB diagnostics in difficult to diagnose populations such as children, people living with HIV with advanced immunosuppression, extrapulmonary TB, and pregnant women.
- Increased use of prospective cohort studies to validate available TB diagnostic biomarkers, rather than validation through retrospective studies which can lead to increased bias. This includes evaluation of diagnostic biomarkers in multi‐center multi‐country consortia to better encompass heterogeneity in active TB disease states and patient populations.
